# A Cross-Sectional Survey of Biosafety Professionals Regarding Genetically Modified Insects

**DOI:** 10.1177/1535676019888047

**Published:** 2019-12-05

**Authors:** David A. O’Brochta, Willy K. Tonui, Brinda Dass, Stephanie James

**Affiliations:** 1 The Foundation for the National Institutes of Health, USA; 2 Environmental Health Safety Ltd., Kenya

**Keywords:** genetically modified insects, insect containment, gene drive, gene editing, accreditation, risk assessment, biosafety

## Abstract

**Background:**

Genetic technologies such as gene editing and gene drive create challenges for existing frameworks used to assess risk and make regulatory determinations by governments and institutions. Insect genetic technologies including transgenics, gene editing, and gene drive may be particularly challenging because of the large and increasing number of insect species being genetically modified and the degree of familiarity with these organisms and technologies by biosafety officials charged with making containment decisions.

**Methods:**

An anonymous online survey of biosafety professionals was distributed to the membership of ABSA International, a global society of biosafety professionals, to investigate their perspectives on their preparedness to meet these new challenges.

**Results:**

Existing guidance used to make containment decisions for nongenetically modified insects was widely seen as adequate, and most respondents thought the available guidance for making containment decisions for genetically modified insects with and without gene drives was inadequate. Most respondents reported having less confidence in their decisions concerning containment of genetically modified insects compared to decisions involving genetically modified microbes, (noninsect) animals, and plants.

**Conclusions:**

These results reveal a need for additional support for biosafety professionals to improve the quality of and confidence in containment decisions regarding genetically modified insects with and without gene drive. These needs might be addressed by increasing training, updating existing guidance, creating new guidance, and creating a third-party accreditation entity to support institutions. Sixty percent of the respondents said they either would or might use a voluntary third-party accreditation service to support insect containment decisions.

## Introduction

Genetic technologies for creating transgenic organisms and precisely modifying genomes are being developed and are evolving rapidly, as are the number of ways in which these technologies can be applied to address problems in medicine, public health, and agriculture. These technologies and their applications create challenges for government and institutional decision makers relying on existing frameworks to assess risk and make regulatory and containment determinations.^1-5^ This is particularly true for decision makers determining containment requirements for genetically modified insects. To date, more than 40 species of insect already have been genetically transformed using transposon-based gene vectors,^6-13^ and 26 species have successfully undergone germline genetic modification using CRISPR/Cas9 gene editing technology, a technology that has only been available since 2013.^14-19^ The rapid pace with which insects are being genetically modified is expected to continue, and as methods improve for using these genetic technologies in a growing number of insect species, the pace with which genetically modified insects are developed will accelerate.

Genetic technologies are being widely adopted by insect scientists because they enable difficult questions in insect biology to be addressed as well as the development of new insect control technologies such as those based on gene drive.^20^ Gene drive technologies are a powerful set of new genetic technologies that are now easily assembled in the laboratory and introduced into insect genomes. Synthetic gene drive systems have the properties of selfish genetic elements, capable of being preferentially transmitted to the next generation during sexual reproduction.^21^ These systems could serve as platforms on which to build specific, sustainable, self-propagating insect population-suppression and -modification technologies capable of addressing some of the world’s most intractable public health and agricultural problems caused by insects.^22^ Insects containing these technologies present new containment challenges since active synthetic gene drive systems are designed to persist and increase in frequency in target populations as well as spread under certain conditions to other conspecific populations.^23-26^ In some cases, such as so-called threshold-independent gene drives, spread of the gene drive construct can be initiated with the release of a single gene drive–containing insect.

The expanded use of insect genetic technologies, including gene drives, has elicited concerns within research communities and among environmental and biological safety experts that escape or release of genetically modified and gene drive–containing insects, especially threshold-independent drives, could result in unintended changes to the environment and significantly erode public trust in the research.^24,25,27-31^ Recently, recommendations for the laboratory containment and management of synthetic gene drive systems in arthropods were published in which the authors recognized that special considerations regarding containment and insectary management may be needed for this class of genetically modified organisms.^23,26,32^ Similarly, a large multidisciplinary working group of scientists and other professionals developed recommendations for the safe and ethical testing of synthetic gene drive–containing mosquitoes intended for use as public health tools to reduce or eliminate mosquito-transmitted diseases such as malaria and dengue fever. This working group concluded that international harmonization of standards for the minimum containment requirements for gene drive–containing mosquitoes would be beneficial to researchers, developers, and containment decision makers.^33^ Similarly, in the report *Editing Biosecurity*, the authors identified a need for improving oversight of gene drive research and development and suggested that this might include updating standards for research and development as well as enhancing the creation and dissemination of best practice guidance coming from research communities and professional societies.^34^

Given the rapidly evolving use of insect genetic technologies and the challenges they present, the perspectives of biosafety professionals and other front-line containment decision makers regarding their preparedness to meet these challenges are of interest since they are well placed to identify gaps and needs that should be addressed. Here we report the results of a survey of biosafety professionals experienced in dealing with genetically modified insects in which they were asked about sources of guidance used in making risk assessments and containment recommendations, the adequacy of that guidance, their confidence in making insect-related containment and biosafety decisions as well as that of their institutional biosafety committee when considering projects involving genetically modified insects, including those containing gene drives. Respondents were also asked about their use of neutral third-party accreditation services in general and the likelihood they would consider voluntarily using a third-party accreditation entity to support their work with genetically modified insects. The results revealed areas in which application and harmonization of regulations and guidelines for the containment of genetically modified insects could be enhanced to increase confidence both within and outside institutions that insect genetic technologies are being managed safely and responsibly. There was clear evidence that institutional biosafety professionals would benefit from additional support when dealing with genetically modified insects.

## Methods

An anonymous online survey using a commercial survey tool (Wufoo, https://www.wufoo.com/) and titled *A Biosafety Needs Assessment—Genetically Modified & Gene Drive–Containing Insects* was created consisting of 5 parts with a total of 25 questions and requiring approximately 10 minutes to complete (Supplemental File 1). In collaboration with ABSA International, the Association for Biosafety and Biosecurity (https://absa.org/), an invitation to participate in the online survey was delivered to its members via email, followed by a second invitation approximately 2 weeks later.

The survey was designed to solicit responses that revealed (1) the responsibilities of respondents and their experience with genetically modified insects, (2) a partial inventory of insects housed at the respondents’ institutions, (3) the sources used by respondents for guidance in assessing risk and containment requirements for insects (nongenetically modified, genetically modified with and without gene drives), (4) the level of confidence of respondents and their institutional biosafety committees in assessing risk and containment requirements for genetically modified insects with and without gene drives, and (5) the familiarity of respondents with third-party laboratory accreditation services and their willingness to use such services that specialized in insect biosafety and containment.

The data were downloaded from Wufoo.com to Microsoft Excel and processed by removing (1) duplicate entries, (2) those who indicated they were not responsible for biosafety compliance, (3) those whose current and prior institutions did not maintain insects or who were uncertain about the insect status of both their current and previous positions and therefore unlikely to have relevant experiences with genetically modified insects, and (4) those who did not complete the entire survey.

## Results

### Sample Size

An invitation to participate in the survey was emailed to the approximately 1700 members of ABSA International. A total of 145 responses were received, which after removing duplicate responses, responses from those who indicated they were not responsible for biosafety compliance, responses from those whose current and prior institutions did not maintain insects, and responses from those who did not complete the entire survey, resulted in 76 unique completed surveys from respondents with relevant experiences and who had responsibilities that included laboratory safety and/or compliance. Based on the respondents’ self-identification, there were 56 biosafety officers, 7 manager/director/administrators, 5 academic titles, 2 consultants, 2 entomologists, and 4 other. All but 9 of the respondents were in the United States (Supplemental File 2).

### Experience with Insects

**Figure 1.**
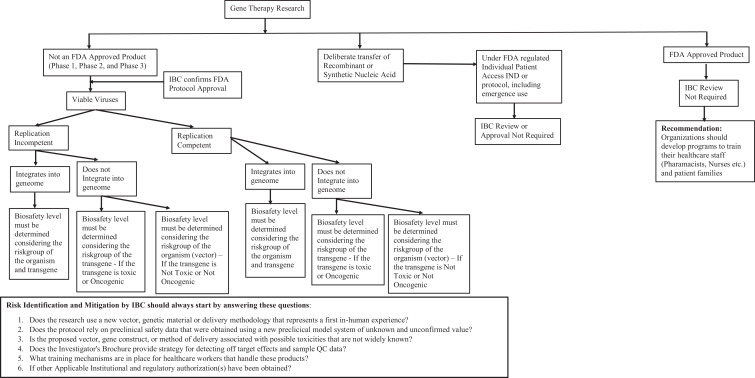
(A) Types of insects and (B) associated risk group of agents.^35^ n = 76.

All data used in the analysis were from respondents that are currently associated with institutions that have insect containment facilities or had been associated with such an institution in the past. Seventy-four percent of the respondents were associated with institutions that contained transgenic insects, whereas 18% of the respondents reported the presence of gene drive–containing insects at their institution (Figure 1A). Collectively, respondents reported 50 or more species of nongenetically modified insects, approximately 20 species of genetically modified insects, and 4 species of gene drive–containing insects. Respondents were also asked to identify risk group of agents associated with insects housed at their institutions^35^ (Figure 1B).

### Use of Risk and Containment Guidance

**Figure 2.**
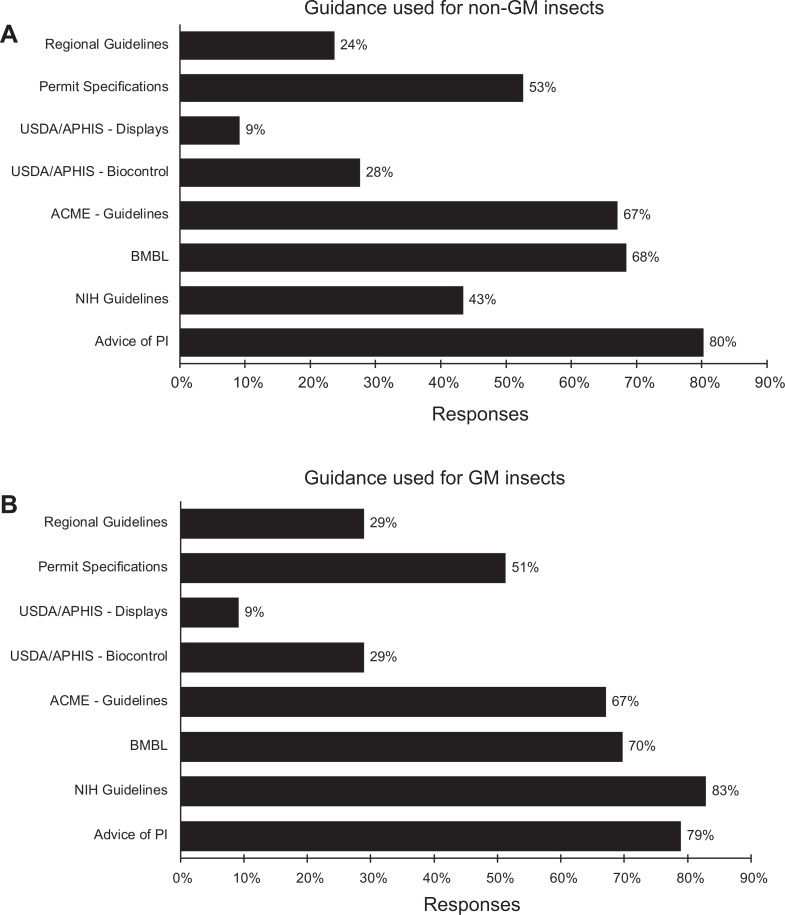
Guidance documents used in assessing risks and containment requirements for (A) nongenetically modified insects and (B) genetically modified insects. n = 76. ACME–Guidelines, arthropod containment guidelines^36^; Advice of PI, advice and recommendation of the principal investigator; BMBL, *Biosafety in Microbiological and Biomedical Laboratories* ^35^; *NIH Guidelines, NIH Guidelines for Research Involving Recombinant or Synthetic Nucleic Acid Molecules* ^39^; Permit Specification, specifications and conditions associated with a permit; Regional Guidelines, national/regional/state/local requirements or guidelines; USDA/APHIS–Biocontrol, *Containment Guidelines for Nonindigenous, Phytophagous Arthropods and Their Parasitoids and Predators* ^38^; USDA/APHIS–Displays = *Containment Guidelines for the Receipt, Rearing and Display of Nonindigenous Arthropods in Zoos, Museums, and Other Public Displays* ^37^.

Respondents were presented with a list of 8 sources of guidance potentially relevant in considering risks and containment requirements associated with insect species along with space for them to provide additional information about other sources of guidance. The 8 sources of guidance were: *Arthropod Containment Guidelines* of the American Committee of Medical Entomologists; *Biosafety in Microbiological and Biomedical Laboratories* (*BMBL*); *USDA Containment Guidelines for Nonindigenous, Phytophagous Arthropods and their Parasitoids and Predators*; *USDA Containment Guidelines for the Receipt, Rearing and Display of Nonindigenous Arthropods in Zoos, Museums, and Other Public Displays; NIH Guidelines for Research Involving Recombinant or Synthetic Nucleic Acid Molecules*; specifications and conditions associated with a permit; national/regional/state/local requirements or guidelines; and advice and recommendation of the principal investigator.^35-39^ There was little difference between responses regarding genetically modified and nongenetically modified insects with the exception that the NIH guidelines on recombinant DNA were used much more frequently when considering genetically modified insects (43% vs 83%; Figure 2). Risk and containment assessments relied heavily on the advice and recommendations of the principal investigator. Approximately 80% of the respondents reported reliance on the principal investigator in their decision-making process, whereas the *Arthropod Containment Guidelines* from the American Committee of Medical Entomologists of the American Society of Tropical Medicine and Hygiene was reportedly used by 67% of the respondents when considering genetically modified insects (Figure 2).

**Figure 3.**
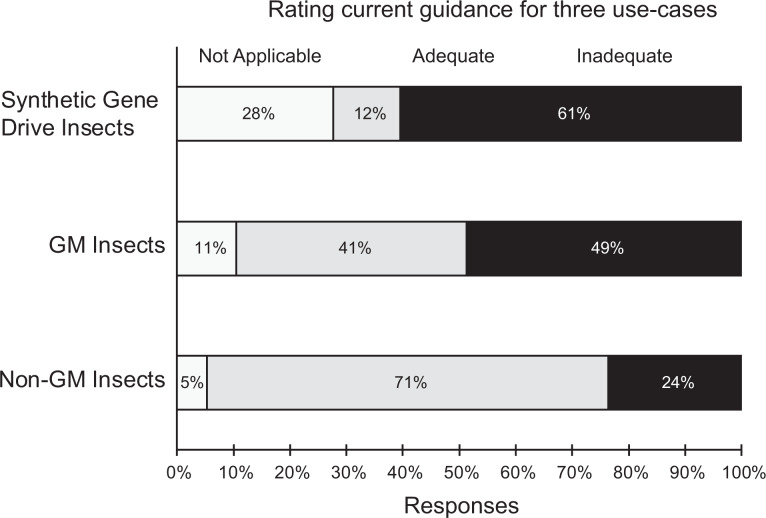
Ratings of currently available guidance documents consulted by respondents when assessing risks and containment requirements for nongenetically modified insects, genetically modified insects, and genetically modified insects with synthetic gene drives. Respondents had the option of “not applicable” to accommodate possible situations where the respondent was not making decisions about either nongenetically modified insects, genetically modified insects, or genetically modified insects with gene drive and therefore was not making use of existing guidance for those applications.

When asked whether the guidance documents they rely on for insect risk assessment and containment decisions were adequate or inadequate, respondents’ responses varied depending on the transgenic genetic status of the insects. Three quarters (75%) of respondents thought existing guidance for nongenetically modified insects was adequate (Figure 3). When considering genetically modified insects, only 46% thought existing guidance was adequate (Figure 3), whereas only 16% of the respondents thought existing guidance was adequate when considering risk and containment of genetically modified insects containing gene drives (Figure 3).

### Confidence and Experience of Decision Makers

**Figure 4.**
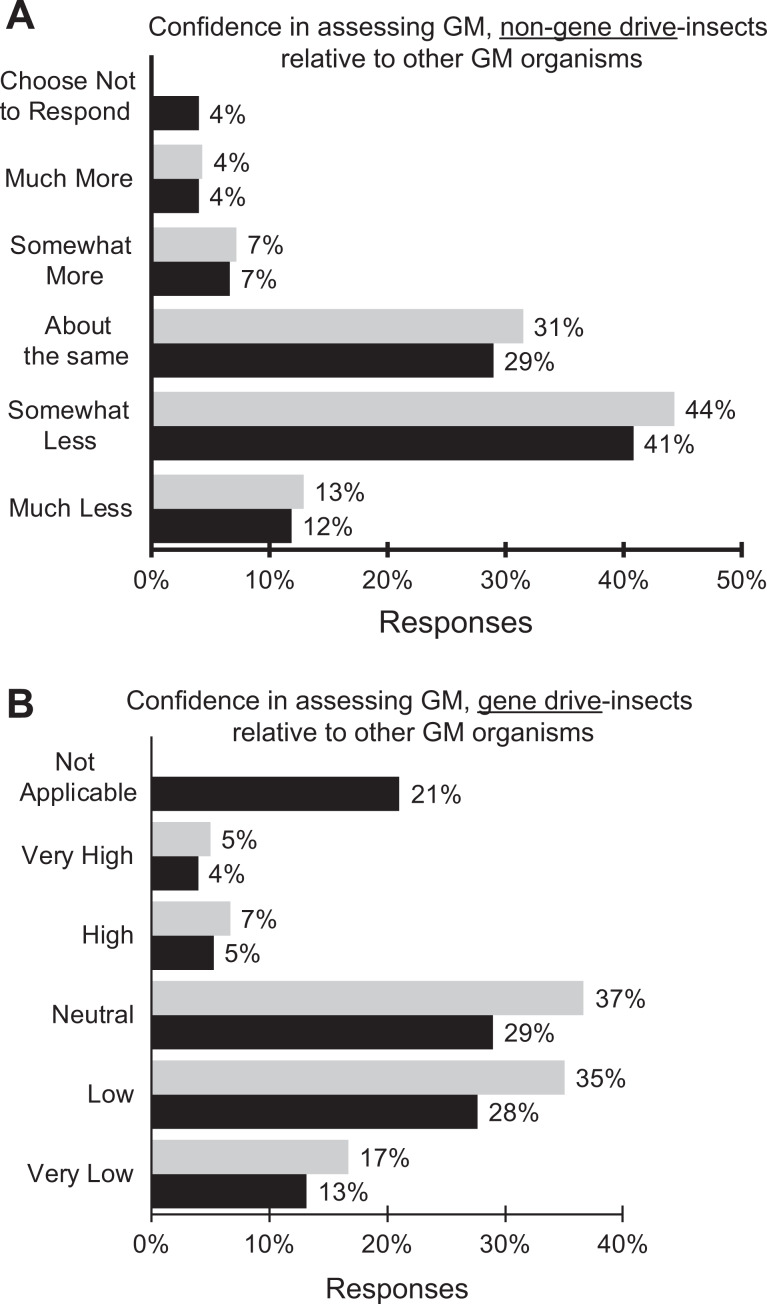
Confidence of respondents in assessing the risks and containment requirements associated with (A) genetically modified insects without gene drive compared to other genetically modified organisms (eg, microbes, animals, plants); black = all responses (n = 76) and grey = only applicable responses (n = 73); (B) genetically modified insects with synthetic gene drive compared to other genetically modified organisms (eg, microbes, animals, plants); black = all responses (n = 76) and grey = only applicable responses (n = 60). Respondents had the option of responding “not applicable” to accommodate the possibility that they did not have experience with genetically modified insects with or without gene drive.

Fifty-seven percent of the respondents rated their level of confidence in assessing risks and containment requirements for laboratories working with genetically modified insects as much less or significantly less confident compared to when they were making similar assessments of other genetically modified organisms such as microbes, animals, and plants (Figure 4A). About the same level of confidence (52%) was reported when considering gene drive–containing insects (Figure 4B).

**Figure 5.**
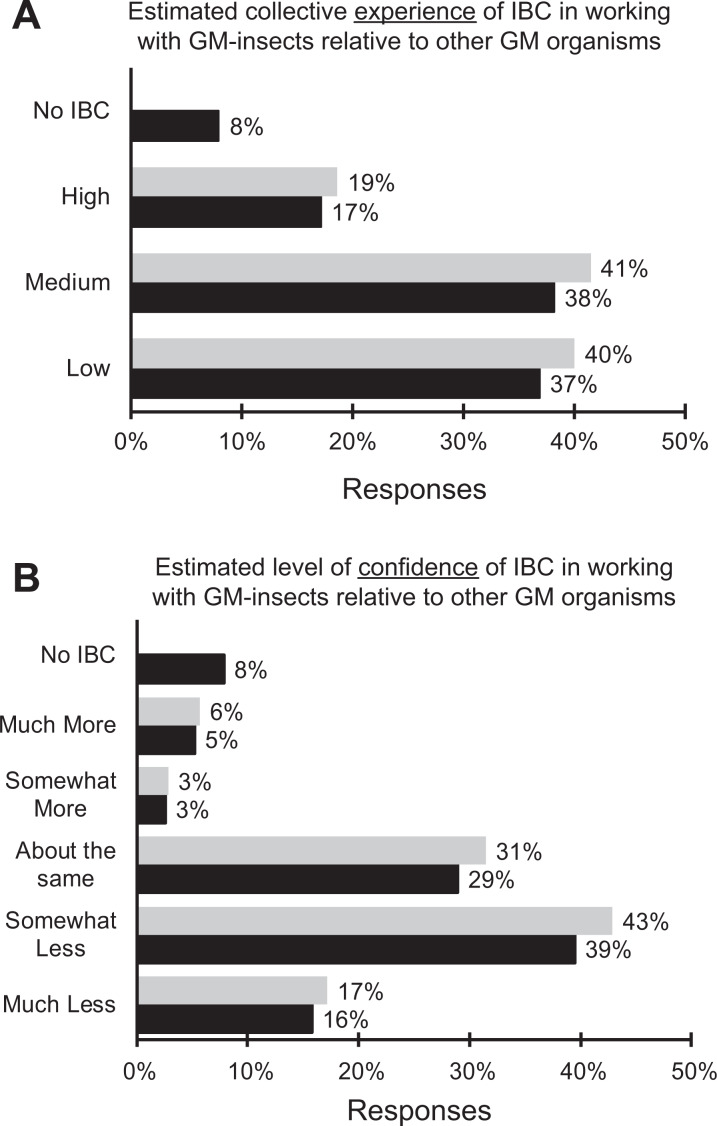
Respondents’ estimation of the level of their institutional biosafety committees’ (A) collective experience in assessing risks and containment requirements for laboratories working with genetically modified insects compared to other genetically modified organisms (eg, microbes, animals, plants); black = all responses (n = 76) and grey = only those with institutional biosafety committees (n = 70); (B) level of confidence in assessing risks and containment requirements for laboratories working with genetically modified insects compared to other genetically modified organisms (eg, microbes, animals, plants), black = all responses (n = 76) and grey = only those with institutional biosafety committees (n = 70).

When asked to estimate the collective level of experience of their institutional biosafety committee (IBC) in assessing risks and containment requirements for laboratories working with genetically modified insects compared to those working with other genetically modified organisms, 40% rated the experience of their institutional biosafety committee as low, whereas 19% rated their IBC’s level of experience as high (Figure 5A).

Similarly, the IBC’s level of confidence in assessing risks and containment requirements of laboratories working with genetically modified insects compared to those working with other genetically modified organisms was estimated to be much less or somewhat less by 60% of the respondents whose institutions had a biosafety committee (Figure 5B).

### Use of Third-Party Accreditation Services

**Figure 6.**
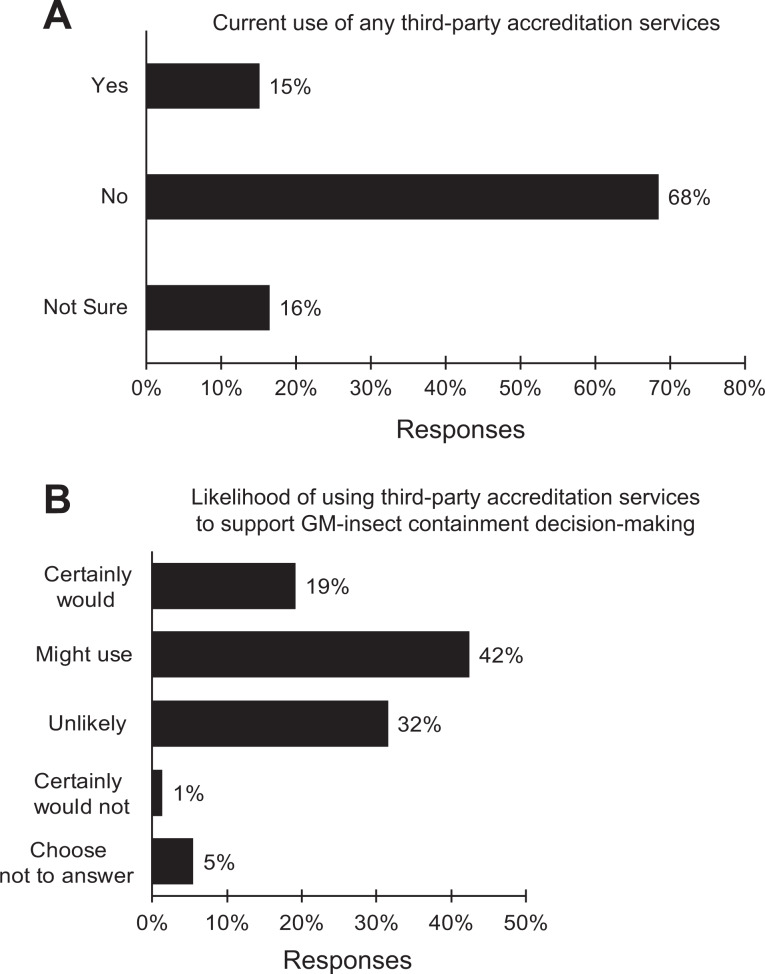
Responses to inquiries about third-party accreditation including (A) current use of third-party services of any kind and (B) likelihood of using a third-party service to support risk assessment and containment decision making and management; black = all responses (n = 73).

A large majority, 68%, of the respondents reported having no experience using third-party conformity/assessment entities for any of their official responsibilities, whereas 15% reported having experience with such entities (Figure 6A). When asked how likely it would be that they would use a voluntary, neutral third-party consulting or accrediting entity to assist them in assessing risk, containment requirements, and management practices of laboratories housing genetically modified insects with or without synthetic gene drives assuming cost was not an issue, 20% of the 69 respondents who chose to answer this question said they certainly would use such services, and 45% said they might use such services. Only 1% said they certainly would not use a third-party accreditation service for the purpose described (Figure 6B).

## Discussion

Advances in the development of insect genetic technologies, including germline transformation and gene editing, are democratizing insect genetic modification, resulting in their increased application in basic and applied insect research. Interest is growing in the potential application of genetic biocontrol strategies for insect disease vectors and agricultural pests. Novel synthetic gene drives with exceptional capabilities of persisting and spreading within natural populations and resembling homing endonucleases found in some organisms such as yeasts can now be readily assembled in the laboratory using RNA-guided DNA endonucleases such as those from the CRISPR/Cas9 gene editing system.^21,22^ Other synthetic gene drives unrelated to homing endonucleases that use different mechanisms and strategies for their effective self-propagation in populations have also been successfully assembled and tested in the laboratory.^22^ The accelerated pace with which scientists are now adopting transgenic technologies is presenting challenges to investigators, institutional biosafety officers, and institutional biosafety committees as they consider the containment requirements of novel genetically modified insects.

For these reasons, we conducted a survey of institutional biosafety officials and experts who are members of ABSA International to obtain their opinions regarding the adequacy of existing guidelines relevant to their making insect containment decisions.

In the United States, containment standards for insects currently used in research are largely described in voluntary guidelines, most of which have very little specific information on transgenic insects. Furthermore, containment facilities are usually not inspected by central authorities such as the National Institutes of Health, the Centers for Disease Control, or the United States Department of Agriculture (USDA). Notable exceptions are USDA-certified quarantine facilities that house and test nonindigenous insects for potential biological control applications and ad hoc USDA inspections of containment facilities that are requesting permits to receive certain genetically modified insects through international importation or interstate movement.

Respondents reported relying heavily on the advice of a project’s principal investigator in their decision making. Less than 70% reported using the *Arthropod Containment Guidelines* of the American Committee of Medical Entomologists, which were written to cover vectors of human pathogens/parasites and included some consideration of transgenic insect vectors.^36^ The reference *Biosafety in Microbiological and Biomedical Laboratories*, which does not explicitly cover transgenic insects, was consulted by about the same fraction of respondents (∼70%).^35^ When dealing with genetically modified insects, 83% of the respondents reported using the *NIH Guidelines for Research Involving Recombinant or Synthetic Nucleic Acid Molecules*.^39^ Section III D-4 of the *NIH Guidelines* cautions that “special care should be used in the evaluation of containment conditions of some experiments with transgenic animals,” but no specific guidance is provided other than suggesting increased containment when the transgenic host animal has “undesirable traits.” Under these circumstances, appropriate containment is expected to be determined by IBCs (Section III D-4-b).

The results of this survey revealed that respondents mostly agreed that existing guidance used by biosafety officials and IBCs is largely inadequate for their evaluation of genetically modified insects, including those containing synthetic gene drives. The perceived inadequacies of existing guidance and the estimated low levels of experience and confidence of IBCs in assessing risk and containment requirements of projects involving genetically modified insects likely result in great variability in containment standards for genetically modified insects among institutions.

Satisfying the needs identified in this survey could involve any number of strategies. Updating applicable standards and best practices guidance documents has been recommended.^34^ Concerned researchers and relevant professional societies may have an important role to play here, as previous efforts have already demonstrated.^24,25,40^ For example, the Arthropod Containment Guidelines written by the American Committee of Medical Entomology within the American Society of Tropical Medicine and Hygiene were drafted to address a perceived lack of guidance for arthropod vectors of human and other animal pathogens and parasites.^36^ These guidelines could form the basis for an enlarged set of guidelines that encompass all insects and specifically address insect genetic technologies such as gene drive. International organizations could also play a key role in developing and promulgating appropriate guidance.^41^

Increased training could improve understanding by researchers and biosafety professionals of insect genetic technologies and familiarity with existing best practices within the insect research community. Online learning modalities have enhanced delivery of biosafety knowledge and training and would be well suited to satisfying some of the needs revealed in this study.

Finally, an external entity with experts familiar with relevant regulations and guidance documents as well as the norms and best practices of the research community could serve as a periodic resource for institutions to support their efforts to meet their biosafety objectives. Third-party accreditations can be voluntary peer assessments intended to enhance quality, ensure that prescribed standards and guidelines are being followed, assure funders and supporters that every effort is being made to conduct research responsibly, and engender public confidence in researchers, their institutions, and their results. AAALAC International (Association for Assessment and Accreditation of Laboratory Animal Care) is an example of a voluntary third-party accreditation service that has served to harmonize compliance standards and foster public confidence in the commitment of institutions to the responsible conduct of laboratory research involving animals.^42^

In this study, survey participants were asked about their experience with third-party accreditation services and the likelihood of their using such a service that could support their compliance efforts associated with genetically modified insects. Approximately 60% of the survey’s respondents said they either certainly would or might avail themselves of a voluntary third-party accreditation service for insect containment facilities and management practices. As with other third-party accreditation services, this could serve to raise and harmonize transgenic insect containment standards.

Appropriate and consistent containment of genetically modified insects is important not only to protect against possible harms that might result should certain genetically modified insects unintentionally enter the environment but also to protect against the erosion of public trust in scientists and institutions as well as possible legal repercussions that could occur following the unintended release of genetically modified insects. Such an erosion in public trust, reputational harm to individuals and institutions, as well as possible legal and financial liability could significantly impede research and development involving the use of insect genetic technologies and their applications to improve public health and food security. This study revealed how biosafety professionals are being challenged by insect genetic technologies and that existing resources may need to be augmented to further support decision making.

## Supplemental Material

Supplemental Material, Biosafety_Survey_2018_For_Paper_Redacted - A Cross-Sectional Survey of Biosafety Professionals Regarding Genetically Modified Insects Click here for additional data file. Supplemental Material, Biosafety_Survey_2018_For_Paper_Redacted for A Cross-Sectional Survey of Biosafety Professionals Regarding Genetically Modified Insects by David A. O’Brochta, Willy K. Tonui, Brinda Dass and Stephanie James in Applied Biosafety

Supplemental Material, Supplement_2 - A Cross-Sectional Survey of Biosafety Professionals Regarding Genetically Modified Insects Click here for additional data file. Supplemental Material, Supplement_2 for A Cross-Sectional Survey of Biosafety Professionals Regarding Genetically Modified Insects by David A. O’Brochta, Willy K. Tonui, Brinda Dass and Stephanie James in Applied Biosafety

## References

[1] Tomlinson T A CRISPR future for gene-editing regulations: a proposal for an updated biotechnology regulatory system in an era of human genomic editing. Fordham Law Rev. 2018;87(1):437–483. 30296034

[2] Fritsche S Poovaiah C MacRae E, et al. A New Zealand perspective on the application and regulation of gene editing. Front Plant Sci. 2018;9:1323. 30258454 10.3389/fpls.2018.01323 PMC6144285

[3] Whelan AI Lema MA A research program for the socioeconomic impacts of gene editing regulation. GM Crops Food. 2017;8(1):74–83. 28080208 10.1080/21645698.2016.1271856 PMC5592976

[4] Waltz E Gene-edited CRISPR mushroom escapes US regulation. Nature. 2016;532(7599):293. 27111611 10.1038/nature.2016.19754

[5] Kim J Kim JS Bypassing GMO regulations with CRISPR gene editing. Nat. Biotechnol. 2016;34(10):1014–1015. 27727209 10.1038/nbt.3680

[6] Handler AM O’Brochta DA Transposable elements for insect transformation In: Gilbert LI, ed. Insect Molecular Biology and Biochemistry. San Diego: Academic Press; 2012:90–133.

[7] Chu F Klobasa W Wu P, et al. Germline transformation of the western corn rootworm, *Diabrotica virgifera virgifera*. Insect Mol Biol. 2017;26(4):440–452. 28397990 10.1111/imb.12305

[8] Xu Q Guerrero FD Palavesam A Pérez de León A Use of electroporation as an option to transform the horn fly, *Haematobia irritans*: a species recalcitrant to microinjection. Insect Sci. 2016;23(4):621–629. 25645001 10.1111/1744-7917.12207

[9] Genc H Schetelig MF Nirmala X Handler AM Germline transformation of the olive fruit fly, *Bactrocera oleae* (Rossi) (Diptera: Tephritidae), with a *piggyBac* transposon vector. Turkish Journal of Biology. 2016;40(4):845–855.

[10] Caroti F Urbansky S Wosch M Lemke S Germ line transformation and in vivo labeling of nuclei in Diptera: report on *Megaselia abdita* (Phoridae) and *Chironomus riparius* (Chironomidae). Dev Genes Evol. 2015;225(3):179–186. 26044750 10.1007/s00427-015-0504-5 PMC4460289

[11] Schulte C Theilenberg E Muller-Borg M Gempe T Beye M Highly efficient integration and expression of *piggyBac*-derived cassettes in the honeybee (*Apis mellifera*). Proc Natl Acad Sci USA. 2014;111(24):9003–9008. 24821811 10.1073/pnas.1402341111 PMC4066538

[12] Schetelig MF Handler AM Germline transformation of the spotted wing drosophilid, *Drosophila suzukii*, with a *piggyBac* transposon vector. Genetica. 2013;141(4-6):189–193. 23564446 10.1007/s10709-013-9717-6

[13] Liu D Yan SC Liu D Huang Y Tan A Stanley DW Song Q Genetic transformation mediated by *piggyBac* in the Asian corn borer, *Ostrinia furnacalis* (LEPIDOPTERA: Crambidae). Arch Insect Biochem Physiol. 2012;80(3):140–150. 22696097 10.1002/arch.21035

[14] Taning CNT Van Eynde B Yu N Ma S Smagghe G CRISPR/Cas9 in insects: applications, best practices and biosafety concerns. J Insect Physiol. 2017;98:245–257. 28108316 10.1016/j.jinsphys.2017.01.007

[15] Zuo YY Huang JL Wang J, et al. Knockout of a P-glycoprotein gene increases susceptibility to abamectin and emamectin benzoate in *Spodoptera exigua*. Insect Mol Biol. 2018;27(1):36–45. 28753233 10.1111/imb.12338

[16] Xue WH Xu N Yuan XB, et al. CRISPR/Cas9-mediated knockout of two eye pigmentation genes in the brown planthopper, *Nilaparvata lugens* (Hemiptera: Delphacidae). Insect Biochem Mol Biol. 2018;93:19–26. 29241845 10.1016/j.ibmb.2017.12.003

[17] Yang Y Wang YH Chen XE, et al. CRISPR/Cas9-mediated Tyrosine hydroxylase knockout resulting in larval lethality in *Agrotis ipsilon*. Insect Sci. 2018;25(6):1017–1024. 30328670 10.1111/1744-7917.12647

[18] Ohde T Takehana Y Shiotsuki T Niimi T CRISPR/Cas9-based heritable targeted mutagenesis in *Thermobia domestica*: a genetic tool in an apterygote development model of wing evolution. Arthropod Struct Dev. 2018;47(4):362–369. 29908341 10.1016/j.asd.2018.06.003

[19] Cong L Ran FA Cox D, et al. Multiplex genome engineering using CRISPR/Cas systems. Sci. 2013;339(6121):819–823. 10.1126/science.1231143 PMC3795411 23287718

[20] Harvey-Samuel TA Ant T Alphey L Towards the genetic control of invasive species. Biol Invasions. 2017;19(6):1683–1703. 28620268 10.1007/s10530-017-1384-6 PMC5446844

[21] Burt AC Crisanti A Gene drive: evolved and synthetic. ACS Chem Biol. 2018;13(2):343–346. 29400944 10.1021/acschembio.7b01031 PMC5820655

[22] Champer J Buchman A Akbari OS Cheating evolution: engineering gene drives to manipulate the fate of wild populations. Nat Rev Genet. 2016;17(3):146–159. 26875679 10.1038/nrg.2015.34

[23] van der Vlugt CJBB Brown DD Lehmann K Leunda A Willemarck N A framework for the risk assessment and management of gene drive technology in contained use. Applied Biosafety. 2018;23(1):25–31.

[24] Adelman Z Akbari O Bauer J, et al. Rules of the road for insect gene drive research and testing. Nat Biotechnol. 2017;35(8):716–718. 28787415 10.1038/nbt.3926 PMC5831321

[25] Akbari OS Bellen HJ Bier E, et al. Safeguarding gene drive experiments in the laboratory. Science. 2015;349(6251):927–929. 26229113 10.1126/science.aac7932 PMC4692367

[26] Benedict MQ Burt A Capurro ML, et al. Recommendations for laboratory containment and management of gene drive systems in arthropods. Vector-Borne and Zoonotic Dis. 2018;18(1):2–13. 10.1089/vbz.2017.2121 PMC5846571 29040058

[27] Kuiken T DARPA’s synthetic biology initiatives could militarize the environment. Slate. 2017 http://www.slate.com/articles/technology/future_tense/2017/05/what_happens_if_darpa_uses_synthetic_biology_to_manipulate_mother_nature.html. Accessed November 19, 2019.

[28] National Academies of Sciences E, Medicine. Gene Drives on the Horizon: Advancing Science, Navigating Uncertainty, and Aligning Research with Public Values. Washington, DC: The National Academies Press; 2016. 27536751

[29] Garthwaite J. U.S. military preps for gene drives run amok. Scientific American. 2016 https://www.scientificamerican.com/article/u-s-military-preps-for-gene-drives-run-amok/. Accessed November 19, 2019.

[30] Civil Society Working Group on Gene Drives. Common call for a global moratorium on genetically-engineered gene drives: SynBiowatch.org; 2016 http://www.synbiowatch.org/wp-content/uploads/2016/12/CBD-Gene-Drive-Sign-on-Letter-English.pdf. Accessed November 19, 2019.

[31] Civil Society Working Group on Gene Drives. The case for a global moratorium on genetically-engineered gene drives: SynBiowatch; 2016 http://www.synbiowatch.org/wp-content/uploads/2016/11/case-for-gene-drive-moratorium.pdf. Accessed November 19, 2019.

[32] Adelman ZN Pledger D Myles KM Developing standard operating procedures for gene drive research in disease vector mosquitoes. Pathog Glob Health. 2017;111(8):436–447. 29350584 10.1080/20477724.2018.1424514 PMC6066849

[33] James S Collins FH Welkhoff PA, et al. Pathway to deployment of gene drive mosquitoes as a potential biocontrol tool for elimination of malaria in sub-Saharan Africa: recommendations of a scientific working group†. Am J Trop Med Hyg. 2018;98(suppl 6):1–49. 10.4269/ajtmh.18-0083 PMC5993454 29882508

[34] Kirkpatrick J Koblentz GD Palmer MJ, et al. Editing Biosecurity: Needs and Strategies for Governing Genome Editing. Palo Alto, CA: Institute for Philosophy and Public Policy, Stanford University; Schar School of Policy and Government; 2018.

[35] Chosewood LC Wilson DE, eds. Biosafety in Microbiological and Biomedical Laboratories. 5th ed Atlanta, GA: U.S. Department of Health and Human Services: Public Health Service, Centers for Disease Control and Prevention, National Institutes of Health; 2009.

[36] ASTMH Containment Guidelines. Arthropod containment guidelines. Vector Borne Zoonotic Dis. 2003;3(2):61–98. 12908960 10.1089/153036603322163448

[37] Animal and Plant Health Inspection Service. Containment guidelines for the receipt, rearing and display of nonindigenous arthropods in zoos, museums, and other public displays. 2002 https://www.aphis.usda.gov/plant_health/permits/downloads/arthropod_biocontrol_containment_guidelines.pdf. Accessed November 19, 2019.

[38] Animal and Plant Health Inspection Service. Containment guidelines for nonindigenous, phytophagous arthropods and their parasitoids and predators. 2002 https://www.aphis.usda.gov/plant_health/permits/downloads/arthropod_biocontrol_containment_guidelines.pdf. Accessed November 19, 2019.

[39] National Institutes of Health. NIH Guidelines for Research Involving Recombinant or Synthetic Nucleic Acid Molecules. Bethesda, DM: National Institutes of Health; 2016.

[40] ASTMH ACME. ASTMH Arthropod Containment Guidelines 3.2. Vector-Borne and Zoonotic Diseases. 2018;19(3):152–173. 10.1089/vbz.2018.2431 PMC6396570 30694736

[41] WHO. Guidance Framework for Testing of Genetically Modified Mosquitoes. Geneva, Switzerland: WHO; 2014.

[42] Gettayacamin M Retnam L AAALAC international standards and accreditation process. Toxicol Res. 2017;33(3):183–189. 28744349 10.5487/TR.2017.33.3.183 PMC5523556

